# Development of Ratiometric Fluorescent Biosensors for the Determination of Creatine and Creatinine in Urine

**DOI:** 10.3390/s17112570

**Published:** 2017-11-08

**Authors:** Hong Dinh Duong, Jong Il Rhee

**Affiliations:** School of Chemical Engineering, Research Center for Biophotonics, Chonnam National University, Yong-Bong Ro77, 61186 Gwangju, Korea; zink1735@gmail.com

**Keywords:** creatine, creatinine, fluorescent sensing membrane, O17-EC membrane, ratiometric calculation

## Abstract

In this study, the oxazine 170 perchlorate (O17)-ethylcellulose (EC) membrane was successfully exploited for the fabrication of creatine- and creatinine-sensing membranes. The sensing membrane exhibited a double layer of O17-EC membrane and a layer of enzyme(s) entrapped in the EC and polyurethane hydrogel (PU) matrix. The sensing principle of the membranes was based on the hydrolytic catalysis of urea, creatine, and creatinine by the enzymes. The reaction end product, ammonia, reacted with O17-EC membrane, resulting in the change in fluorescence intensities at two emission wavelengths (*λ_em_* = 565 and 625 nm). Data collected from the ratio of fluorescence intensities at *λ_em_* = 565 and 625 nm were proportional to the concentrations of creatine or creatinine. Creatine- and creatinine-sensing membranes were very sensitive to creatine and creatinine at the concentration range of 0.1–1.0 mM, with a limit of detection (LOD) of 0.015 and 0.0325 mM, respectively. Furthermore, these sensing membranes showed good features in terms of response time, reversibility, and long-term stability. The interference study demonstrated that some components such as amino acids and salts had some negative effects on the analytical performance of the membranes. Thus, the simple and sensitive ratiometric fluorescent sensors provide a simple and comprehensive method for the determination of creatine and creatinine concentrations in urine.

## 1. Introduction

Creatine, a non-essential nutrient, plays an important role by supplying energy to muscle cells [1]. In the early 1990s, there was a great interest among consumers and researchers concerning therapeutic applications of creatine and benefits of using creatine as a dietary supplement [2]. In healthy adults, creatine levels in biological fluids such as serum or plasma are typically around 0.04–0.15 mM which may rise to above 1 mM under certain pathological conditions such as muscle disorders. Creatine is taken as an ergogenic supplement at a daily dose of 7–30 mM (or up to 140 mM) by athletes to increase their body mass [3]. During oral supplementation, some part of the consumed creatine may be excreted through urine. The difference between the amount of urinary creatine and ingested creatine dose indicates the amount of creatine absorbed by the muscles. Therefore, monitoring creatine levels is important in clinical diagnosis [4]. 

To our knowledge, few studies have been directed toward the development of creatine sensors/biosensors and the commercially available creatine kits are very expensive. Creatine biosensors have been studied for several decades and are based on the hydrolysis of creatine by creatinase and urease to produce ammonia. Ammonia is usually detected by any pH or ion-selective electrodes [5,6]. Alternatively, a second enzyme-catalyzed hydrolysis of creatine mediated by sarcosine oxidase results in the production of hydrogen peroxide (H_2_O_2_) as the end product, which can be detected with a chronoamperometric technique [7,8,9]. Creatine detection has been also performed with flow-injection analysis (FIA) systems combined with immobilized enzyme reactors [10] and more expensive analytical tools such as high-performance liquid chromatography [10,11], capillary electrophoresis [12], and mass spectrometry [13]. However, the use of creatine sensors and other analytical tools is associated with practical issues related to sensitivity, reproducibility, stability, and interferences. 

Creatinine is the end product of creatine metabolism in mammalian cells [1]. Muscular creatine is converted to creatinine, which diffuses out of the cells and is excreted by the kidney into the urine. Therefore, creatinine levels in biological fluids are clinically important for the diagnosis of renal, thyroid, and kidney dysfunctions. The normal concentration of creatinine in human blood ranges from 0.04 to 0.15 mM and these values may rise to 1 mM or more in chronic kidney disease patients. A typical urine sample of an adult contains creatinine at 2.5–23 mM concentration [14]. The ratio of creatinine in human serum and urine sample is an important indicator of the kidney function. Therefore, it is important to monitor creatinine concentrations in biological fluids, including urine, for clinical diagnosis [15]. 

There has been a tremendous improvement in the quality of creatinine biosensors. Some review articles on creatinine biosensors have been published in recent years [16,17,18,19,20]. Creatine and creatinine may interact in a reversible pathway depending on the type of enzymes involved. Therefore, creatine and creatinine biosensors are based on the same principles. Ammonium ion or H_2_O_2_, the final product of the hydrolysis reaction of creatinine catalyzed by one or three enzymes, is often measured to determine creatinine concentrations. Transducers such as amperometry [21,22], potentiometry [23,24], ion-sensitive field effect transistor [25,26,27], or spectrophotometry [28] may be used based on the type of the final reaction products. Among these biosensors, the amperometric method was the first system to be successfully commercialized [17]. 

The use of other biosensors for the determination of creatine and creatinine is still limited. So far, traditional photometric methods based on Jaffe’s reaction have been widely used [29], however, colorimetric methods suffer from interferences during analysis. A diamond paste-based biosensor for creatine and creatinine detection is a rapid and simple sensor, but its measurement ranges are too low, 1–500 pM for creatine and 0.01–100 nM for creatinine [30]. The ratiometric fluorescence method offers some advantages over photometric and amperometric methods in terms of high sensitivity, good selectivity, and reproducibility, while enzyme immobilization allows high specificity, stability, and wide detection range to biosensors. Thus, we have developed ratiometric fluorescence biosensors using immobilized enzymes for the detection of creatine and creatinine. 

Creatine detection is based on the hydrolysis of urea and creatine catalyzed by two enzymes, urease and creatinase, to produce ammonia in the following reactions:

$$\mathrm{Creatine}+H_{2}O\;\overset{\mathrm{Creatinase}}{\to }\;\mathrm{Urea}+\mathrm{Sarcosine}$$

$$\mathrm{Urea}+H_{2}O\;\overset{\mathrm{Urease}}{\to }\;NH_{4}{}^{+}+CO_{2}$$

The enzyme creatinine deiminase catalyzes the conversion of creatinine to ammonia and *N*-methylhydantoin, as depicted in the following reaction:

$$\mathrm{Creatinine}+H_{2}O\;\overset{\mathrm{Creatinine}\;\mathrm{deiminase}}{\to }\;NH_{4}{}^{+}+N\text{-}\mathrm{methylhydantoin}$$

Ratiometric fluorescence biosensor is based on a fluorescent sensing membrane, which is a double-layer membrane of oxazine 170 perchlorate (O17) and ethylcellulose (EC) as an optical transducer and a layer of enzymes entrapped in the matrix of ethylcellulose (EC) and polyurethane hydrogel (PU) [31,32].

**Scheme 1:**
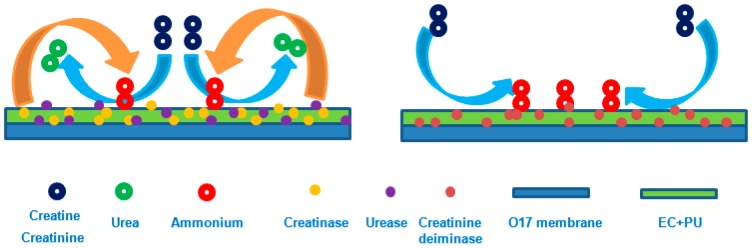
Structure of the creatine sensor (**left**) and creatinine sensor (**right**).

In this study creatine- and creatinine-sensing membranes were prepared (Scheme 1) and their properties were investigated. The sensing membranes were also characterized in terms of their response to creatine and creatinine using ratiometric calculation. These creatine and creatinine biosensors were evaluated for their ability to determine the concentration of creatine and creatinine dissolved in artificial urine solution (AUS).

## 2. Materials and Methods

### 2.1. Materials

Oxazine 170 perchlorate (O17), ethylcellulose (EC), urease (59,400 U/g solid, from *Canavalia ensijormis* (Jack bean)), creatinase (15 U/mg solid, from *Actinobacillus* sp.), creatinine deiminase (20 U/mg solid, *microbial*), creatine, creatinine, glycine, histidine, phenylalanine, tryptophan, and urea were purchased from Sigma-Aldrich Chemical Co. (Seoul, Korea). Tris buffer was obtained from USB Co. (Cleveland, OH, USA) and PU from AdvanceSource Biomaterials Co. (Willmington, MA, USA). Other analytical-grade chemicals such as sodium phosphate, potassium phosphate, sodium chloride, potassium chloride, sodium hydroxide, hydrochloric acid, sodium bicarbonate, magnesium sulfate, sodium sulfate, and calcium chloride were used without further purification.

### 2.2. Preparation of Creatine- and Creatinine-Sensing Membranes

We prepared O17-EC membranes as previously described [31] by mixing O17 stock (15 µL, 2 mg/mL) with EC (300 µL, 10 wt %) in ethanol. The mixture was incubated for 4 h at room temperature and coated on the bottom of each well of a 96-well microtiter plate (NUNC Co. Copenhagen, Denmark). O17-EC membrane was dried at 60 °C for 12 h. In the next step, 20 µL of the mixture of 10 wt % EC and 10 wt % PU in ethanol and water (9:1 in v/v%) was coated onto O17-EC membrane, followed by the addition of the solution of a given amount of urease and creatinase dissolved in 10 mM phosphate buffer (pH 7.4). The enzymes were entrapped in the polymer matrix of EC and PU and immobilized over O17-EC membrane. The creatine-sensing membrane immobilized with enzymes was incubated for 24 h at 4 °C. Surface morphologies of O17-EC and creatine-sensing membrane were identified by atomic force microscopy (AFM). The preparation of the creatinine-sensing membrane was similar to that of the creatine-sensing membrane. Only one enzyme, creatinine deiminase, dissolved in 10 mM phosphate buffer (pH 7.4) was immobilized on O17-EC membrane. 

### 2.3. Measurements of Creatine and Creatinine

Concentrations of creatine and creatinine for measurements were in the range of 0.1 to 10 mM. Data were collected from the fluorescence intensity of the sensing membranes at two emission wavelengths (*λ_em_* = 565 and 625 nm) with an excitation wavelength of 460 nm (*λ_ex_* = 460 nm). The fluorescence spectra for the detection of creatine and creatinine were measured using a multifunctional fluorescence microtiter plate reader (Safire^2^, Tecan Austria GmbH, Wien, Austria).

#### 2.3.1. Creatine Measurements

The optimization of creatinase amount for immobilization was performed with 3.5, 7, 14, 28, and 42 unit (U) of creatinase and a fixed amount of urease (10 U). The immobilization efficiency of urease and creatinase in the creatine-sensing membrane was calculated by dividing the amount of immobilized enzymes with the total amount of enzymes used for immobilization. The amount of the immobilized urease and creatinase was determined by subtracting the amount of the un-immobilized urease and creatinase from the total amount of the enzymes used. The un-immobilized urease and creatinase were separated from the immobilized urease and creatinase in one well by washing several times with 10 mM phosphate buffer (pH 7.4). The protein values of the washed, un-immobilized urease and creatinase were determined by Bradford method. The kinetic parameters, maximal reaction rate (V_max_) and Michaelis-Menten constant (K_m_), of the enzymes co-immobilized on the supporting material of EC and PU were determined from the Hanes plot using the ratio of fluorescence intensities at *λ_em_* = 565 and 625 nm. The sensitivity of creatine-sensing membranes with different amounts of creatinase was evaluated through the slope value (SI), i.e., the ratio of the fluorescence intensities at two emission wavelengths (*λ_em_* = 565 and 625 nm) with respect to creatine. The reversibility of the creatine-sensing membrane was tested in distilled water and 1.0 mM creatine prepared in Tris buffer. The creatine-sensing membrane was first exposed to distilled water and then to 1.0 mM creatine solution and the fluorescence measurements were performed on the microtiter plate reader at an interval of 30 s. In addition, the effects of pH and temperature on the creatine-sensing membrane were investigated. The creatine-sensing membrane was exposed to 1.0 mM creatine solutions at a pH range of 5.0–9.0. The membrane was also tested at different temperatures (25, 30, 33, 35, 37, and 40 °C) at creatine concentrations of 0.1–10 mM. The long-term stability of the creatine-sensing membrane in the presence of creatine at various concentrations was evaluated through the determination of its repeatability by measuring the fluorescence intensity obtained initially and after 3 months. The interfering effects of some components in urine samples on the creatine-sensing membrane were investigated using 1.0 mM creatine and 0.2 mM of glycine, histidine, phenylalanine, tryptophan, and creatinine as well as their mixture. 

#### 2.3.2. Creatinine Measurements

The optimal amount of creatinine deiminase for immobilization on O17-EC membrane was tested with 2.5, 5, and 7.5 U of creatinine deiminase. The immobilization efficiency of creatinine deiminase in the creatinine-sensing membrane was evaluated with Bradford method, as described to measure the immobilization efficiency of enzymes in part Section 2.3.1. The sensitivity of the creatinine-sensing membrane was also evaluated through SI. The kinetic parameters (V_max_ and K_m_) of the immobilized creatinine deiminase were determined from the Lineweaver-Burk plot based on the ratio of the fluorescence intensities at *λ_em_* = 565 and 625 nm. The reversibility of the creatinine-sensing membrane was examined with 0.1 and 1.0 mM creatinine dissolved in Tris buffer and the fluorescence measurements were performed on a microtiter plate at an interval of 30 s. The effects of pH and temperature on the creatinine-sensing membrane were investigated with 0.4 mM creatinine solution at a pH range of 5.0 to 10.0 and temperature of 30, 33, 35, 37, and 40 °C using creatinine concentrations from 0.1 to 10 mM. The long-term stability of the creatinine-sensing membrane at various creatinine concentrations was evaluated by measuring the fluorescence intensity obtained initially and after 1.5 months. The interference effects of some components on the creatinine-sensing membrane were also investigated using 1.0 mM creatinine and 0.2 mM of glycine, histidine, phenylalanine, tryptophan, urea, and creatinine as well as their mixture. 

#### 2.3.3. Artificial Urine Solution (AUS)

Artificial urine solution containing various concentrations of creatine and creatinine was prepared, and the concentrations of creatine and creatinine were determined with the creatine- and creatinine-sensing membranes, respectively. The AUS comprised 2.5 mM CaCl_2_, 45 mM NaCl, 3.5 mM KH_2_PO_4_, 3.5 mM K_2_HPO_4_, 2.5 mM NaHCO_3_, 1 mM MgSO_4_, 2.5 mM Na_2_SO_4_, and creatine or creatinine at concentration range of 0.1–10 mM and pH 7.2. 

### 2.4. Ratiometric Method

The ratiometric method for creatine and creatinine biosensors was based on the ratio of the fluorescence intensities of the creatine- and creatinine-sensing membranes at an excitation wavelength of 460 nm (*λ_ex_* = 460 nm) and two emission wavelengths (*λ_em_* = 565 nm [FI_565_] and 625 nm [FI_625_]) as follows:

R = FI_565_/FI_625_

### 2.5. Data Analysis

The differences in fluorescence intensity of the sensing membranes at different pH, and temperature values were assessed by one-way analysis of variance (ANOVA). A value of *p* < 0.05 was considered as a statistically significant. Statistical tests were performed using the software InStat (v.3.01, GraphPad Software Inc., San Diego, CA, USA).

## 3. Results and Discussion

### 3.1. Enzyme Immobilization for Creatine-Sensing Membrane

**Figure 1:**
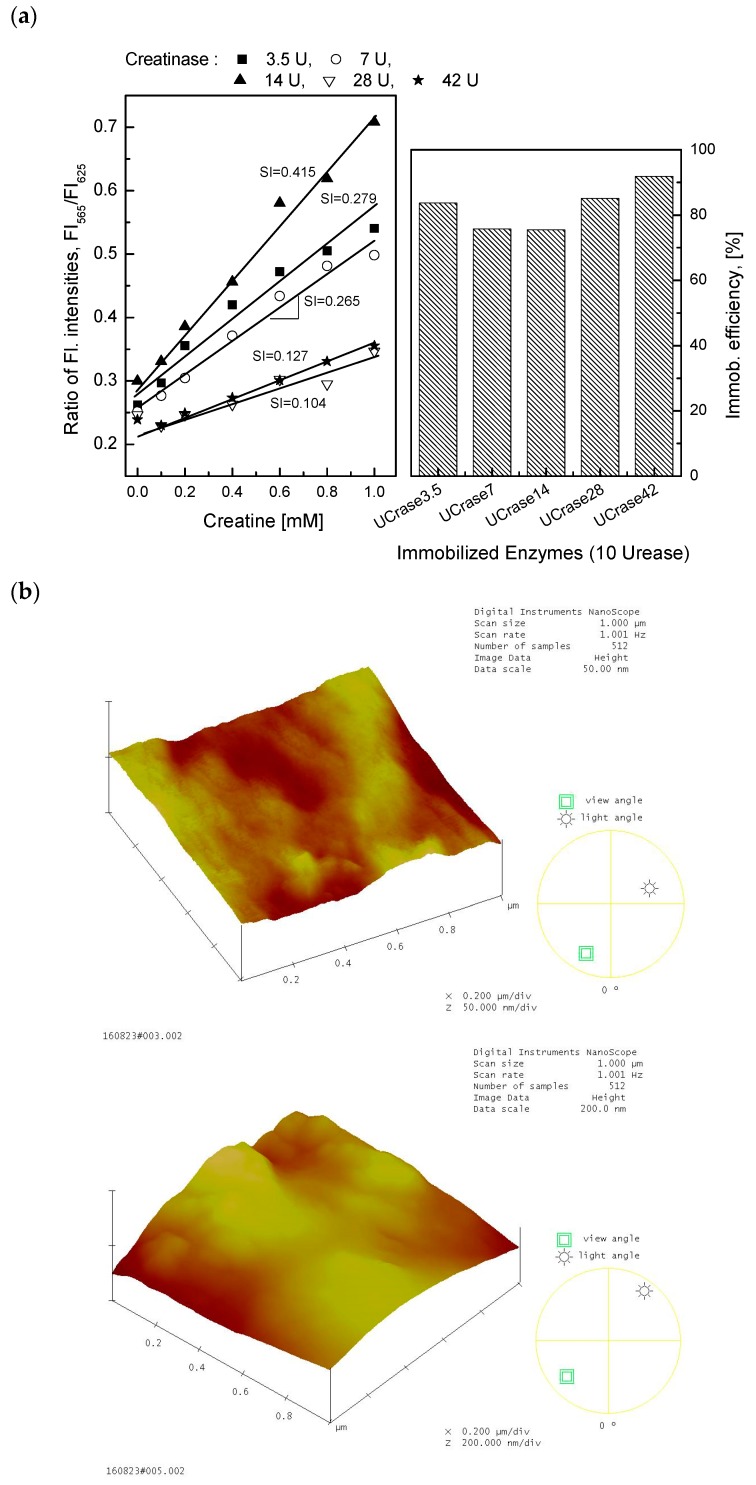
(**a**) The response of the creatine-sensing membrane immobilized with 3.5, 7, 14, 28, and 42 U creatinase and fixed amount (10 U) of urease in the presence of 0.1 to 1.0 mM creatine (**top**). SI presents slope value in the linear detection range of 0.1 to 1.0 mM. The immobilization efficiency for various amounts of creatinase and 10 U of urease (**top**); (**b**) AFM images of O17-EC membrane (**top**) and the second layer containing entrapped enzyme(s) (**below**).

The response of the creatine-sensing membrane in the presence of different amounts of immobilized creatinase is shown in Figure 1a. The creatine-sensing membrane immobilized with 14 U creatinase showed the highest SI (SI_14U_ = 0.415) at creatine concentrations from 0.1 to 1.0 mM. We failed to see any increase in the sensitivity of the membrane at higher amounts (28 and 42 U) of creatinase, as evident from the lower SI at 28 and 42 U (SI_28U_ = 0.104 and SI_42U_ = 0.127) as compared with 14, 7, and 3.5 U creatinase. Results of Bradford protein assay showed that the immobilization efficiency of urease and creatinase on the second layer of the creatine-sensing membrane was very high at all creatinase amounts. The immobilization efficiency for the two enzymes was 83.7%, 75.8%, 75.5%, 85.1%, and 91.9% with 3.5, 7, 14, 28, and 42 U creatinase, respectively. The high immobilization efficiency resulted in the good enzyme-capturing capacity of EC and PU matrix. The immobilization efficiency of urease (10 U) into the matrix of EC and PU was higher than 90% in preliminary studies. Based on the high immobilization efficiency of urease (10 U), the immobilization efficiency for creatinase into EC and PU matrix may be estimated to be higher than 70%. However, the excess of creatinase immobilization into the matrix of EC and PU may obstruct NH_4_^+^ transport in O17-EC membrane. The high density of the enzyme-supporting layer obstructed the passage of NH_4_^+^ in O17-EC membrane below the enzyme layer. Based on these observations, we chose 14 U creatinase to fabricate the creatine-sensing membrane for further work.

The creatine-sensing membrane included two layers. The first layer was O17-EC membrane, while the second layer was the membrane comprising urease and creatinase co-immobilized in the matrix of EC and PU. As seen in AFM images (Figure 1b), O17-EC membrane displayed a smooth surface with a surface mean roughness (Ra) and root mean square roughness (Rq) of 1.07 and 1.343 nm, respectively, whereas the surface of the second layer immobilized with urease and creatinase displayed higher values of Ra and Rq (9.514 and 12.304 nm, respectively). The increase in the roughness of the sensing membrane resulted from the successful immobilization of the enzymes into EC-PU matrix and the subsequent formation of PU hydrogel during the reaction with the aqueous enzyme solution. 

### 3.2. Characterization of the Creatine-Sensing Membrane

**Figure 2:**
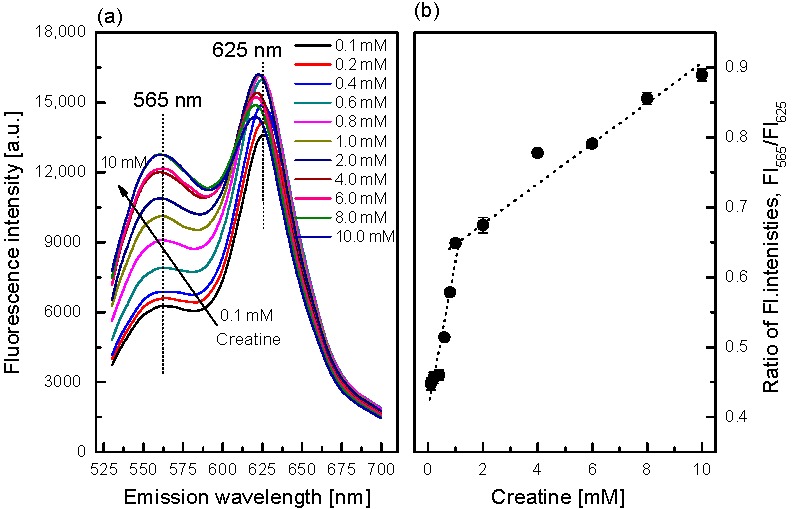
(**a**) Fluorescence emission spectra of the creatine-sensing membrane in the presence of creatine at 0.1 to 10 mM concentration monitored at an excitation wavelength of 460 nm. (**b**) Calibration curve for creatine calculated by the ratio of two fluorescence intensities measured at emission wavelengths of 565 and 625 nm.

As shown in Figure 2, the creatine-sensing membrane was very sensitive to ammonia produced during the hydrolysis reactions of urea and creatine in the presence of urease and creatinase, respectively. The detection range of creatine based on the ratiometric calculation method could be divided into two linear ranges of 0.1–1.0 mM and 1.0–10 mM with high regression coefficient values of *r*^2^_0.1–1.0 mM_ = 0.92 and *r*^2^_1.0–10 mM_ = 0.96, respectively. The relative standard deviation for 1.0 mM creatine was 2.4% (*n* = 7) based on five repetitive measurements, while the detection limit (LOD, S/N = 3) was 0.015 mM. According to the response of O17-EC membrane to different ammonia concentrations [31], the fluorescence intensity of the creatine-sensing membrane would increase at *λ_em_* = 565 nm and decrease at *λ_em_* = 625 nm in the presence of the increasing concentrations of creatine. However, a small increase in the fluorescence intensity was observed at *λ_em_* = 625 nm at low creatine concentrations. This observation may be attributed to the low amount of ammonia, which reacted with O17 dye as per the following equation

NH_4_^+^OH^−^ + H^+^Dye^−^⇔NH_4_^+^Dye^−^ + H_2_O

A large amount of the dye was in its free state. The reaction mechanism of O17 dye and ammonia produced from the catalytic breakdown of creatine was explained in our previous research [32]. Some modifications in the original sensor may result in unexpected observations, as evident from the behavior of the peak at *λ_em_* = 625 nm, which was different from that observed for the original O17 dye. However, the ratiometric method may normalize the change in the fluorescence intensities that are unrelated to the change in the target concentration. Therefore, the values for the ratio of fluorescence intensities at *λ_em_* = 565 and 625 nm corresponded well with creatine concentrations. For fluorescence-based sensors, measurements of fluorescence intensity at a single band edge are known to be problematic for practical applications [33]. The ratiometric fluorescence method is useful for the correction of a variety of analyte-independent factors in fluorescent sensors, wherein the temporal and spatial distribution of the measured fluorescence intensity may typically fluctuate owing to the unequal distribution of fluorophores within the sensor, variation in dynamics of fluorophores in different media, and noise in the measurement system (e.g., variations in the illumination intensity). The self-calibration property of the ratiometric method has led to the development of a wide range of ratiometric fluorescent sensors that provide precise quantitative analysis. Nakata et al. described real-time monitoring of saccharide conversion pathway using a seminaphthorhodafluor-conjugated lectin-based ratiometric fluorescent biosensor [34], whereas Xie et al. exploited the enzymatic reaction of hyaluronidase and hyaluronan bound to two fluorescent dyes for fluorescence quenching and dequenching of these dyes before and after enzyme reaction to obtain the proportion between the ratiometric fluorescence intensity and hyaluronidase level [35]. Other researchers have used ratiometric fluorescence biosensors for the detection of DNA [36] and nitric oxide [37].

Kinetic parameters of creatinase (14 U) and urease (10 U) co-immobilized on the supporting material of EC and PU were evaluated with Michaelis-Menten kinetics using the ratio of fluorescence intensities at *λ_em_* = 565 and 625 nm. An apparent maximal reaction rate (V_max_^app^) of 0.0406 1/min and apparent Michaelis-Menten constant (K_m_^app^) of 3.441 mM were obtained using Hanes plot. The value of K_m_^app^ was greater than that obtained for creatinase immobilized with chitosan-SiO_2_-multiwall carbon nanotubes nanocomposite (K_m_^app^ = 0.58 mM, [38]). K_m_^app^ is usually dependent upon the supporting material and immobilization method. The large value of K_m_^app^ in this work indicates the low affinity of creatinase to EC and PU matrix over EC-O17 membrane and may be associated with the conformation and arrangement of the enzyme during immobilization. The slow reaction rate of the creatine-sensing membrane may be attributed to the retardation of the reaction product (NH_4_^+^OH) toward O17-EC layer through the thick enzyme-immobilized layer. 

The response time of the creatine-sensing membrane was approximately t_95(0.1–0.4 mM)_ = 1 to 2.5 min, t_95(0.6–2 mM)_ = 2.5 to 3.5 min, and t_95(4–10 mM)_ = 0.5 to 1.5 min. Moreover, the addition of PU to the supporting material for enzyme immobilization offered a convenient environment to shorten the transport time of ammonia passing through the enzyme-immobilized membrane to contact O17-EC membrane. The use of PU as a supporting material for enzyme immobilization may improve the response time of the enzyme-immobilized membrane.

**Figure 3:**
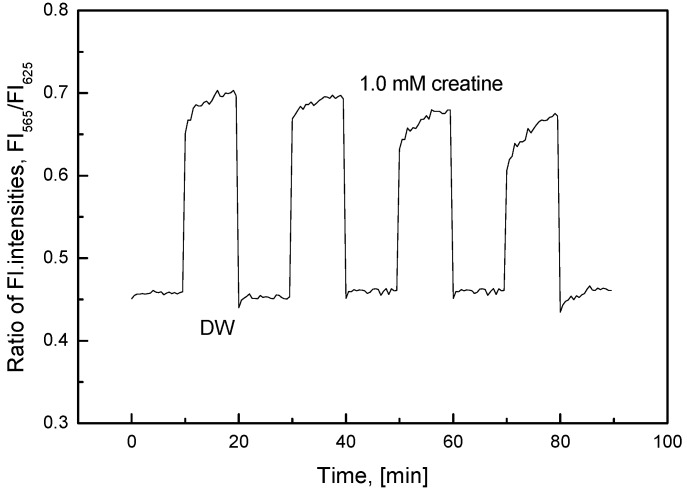
Reversibility of the creatine-sensing membrane in the presence of 1.0 mM creatine and distilled water (DW).

As shown in our previous study [32], O17-EC membrane showed high reversibility despite being coated with a second layer of polymer (e.g., EC) and urease. The fluorescent O17 dye could offer a proton in the formation of NH_4_^+^OH^−^ by the protonation of ammonia in water and react reversibly upon reduction of NH_4_^+^OH^−^. Herein, a sequence of hydrolysis reactions of creatine and urea resulted in the production of ammonia, which reacted with O17-EC membrane and changed the fluorescence intensity of O17 dye. This change was easy to recognize upon repeated exposure of the creatine-sensing membrane to 1.0 mM creatine and distilled water (DW). Figure 3 shows the reversibility of the creatine-sensing membrane as the ratio of the fluorescence intensities at *λ_em_* = 565 and 625 nm. The sensing membrane exhibited a very low relative standard deviation of 0.78% and 2.69% in DW and 1.0 mM creatine, respectively. 

The performance of a biosensor with an enzyme is usually influenced by pH and temperature. pH determines the activity of the enzyme and the consequent efficiency of the catalytic reaction in the biosensor. Similar to pH is the influence of temperature, which may increase or decrease the catalytic reaction rate of the enzyme. The creatine-sensing membrane preferred alkaline (pH range of 7.5 to pH 9.0) to weakly acidic (pH 5.0 to pH 7.0) medium. The ratio of the fluorescence intensities at *λ_em_* = 565 and 625 nm obtained for the creatine-sensing membrane in the presence of 1.0 mM creatine failed to change significantly in the acidic pH range, but considerably increased with an increase in pH from 7.5 to 9.0 (data not shown). Creatine was difficult to dissolve in common aqueous solutions and, hence, was prepared in 10 mM Tris buffer at pH range of 7.5–8.5. Therefore, the creatine-sensing membrane worked well under alkaline conditions induced via ammonia produced from the hydrolysis of creatine and urea or interferences from other alkaline factors. The response of the creatine-sensing membrane to different temperatures was investigated at various creatine concentrations. The temperature range of 25 to 35 °C had no effect on the creatine-sensing membrane in the presence of 0.1 to 10 mM creatine (data not shown). However, the sensitivity of the membrane decreased at high temperatures (37–40 °C) and creatine concentration (1.0–10 mM). However, the sensitivity was unaffected by temperature at low concentrations of creatine (0.1–1.0 mM). Therefore, a temperature of approximately 33 °C was used for creatine measurements to extend the performance of the enzymes as well as the sensitivity of the creatine-sensing membrane for long-term use. 

**Figure 4:**
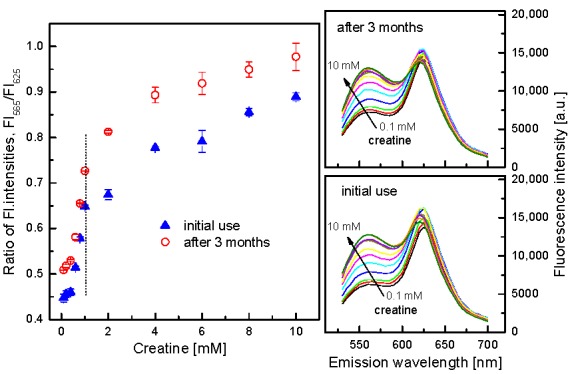
Long-term stability of the creatine-sensing membrane in the presence of creatine 0.1 to 10 mM creatine upon initial use and after 3 months of use (**left**), and two fluorescence emission spectra of the creatine-sensing membrane with respect to creatine concentrations at *λ_ex_* = 460 nm.

The creatine-sensing membrane was tested after 3 months of use and storage; its sensitivity was found to be quite good (Figure 4), as evident from SI of the linear curve with creatine concentration of 0.1 to 1.0 mM (SI_0.1–1.0 mM_) (0.22 at initial use and 0.242 after 3 months of use). Thus, the creatine-sensing membrane including O17-EC layer as a transducer and EC and PU matrix layer immobilized with two enzymes (urease and creatinase) showed excellent stability after long-time use. EC and PU were good supporting materials for the fluorescent dye O17 and enzyme immobilization. The layer of EC and PU matrix prevented enzyme leakage [39], but at the same time created an open environment, which improved the sensitivity of the membrane following ammonia production. The presence of PU in the enzyme-immobilized layer induced softening of the supporting layer for enzyme immobilization, thereby allowing higher amount of enzyme immobilization and offering a wider location for the passing ammonia to react with O7-EC membrane. In addition, we suggest that the covalent binding between the isocyanate groups of PU and amine groups of the enzymes increased the lifetime of the sensor. The increase in the background signal was associated with the increased opacity of the sensing membrane. The change probably occurred owing to the long-term soaking in 10 mM phosphate buffer solution that increased the reflection of the incident light and fluorescence emission during creatine measurements.

**Figure 5:**
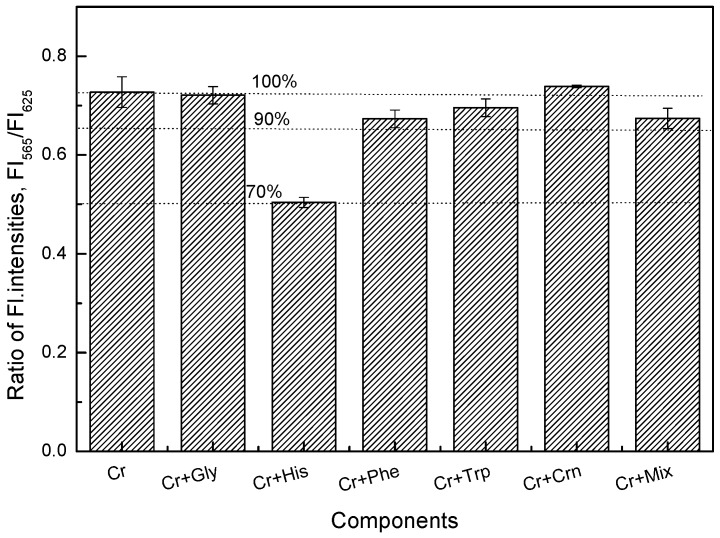
Response of the creatine-sensing membrane to aqueous solutions of 1.0 mM creatine (Cr) and 0.2 mM of each of the following components: Gly; glycine, His; histidine, Phe; phenylalanine, Trp; tryptophan, Crn; creatinine, Mix: mixture of Gly, His, Phe, Trp, and Crn.

The recovery capability of a biosensor may suffer from some interferences existing in the sample. Urine samples may contain several types of carbohydrates, metabolites, and electrolytes, which may interfere with the fluorescent creatine biosensor developed here. According to the results of one-way ANOVA with Tukey-Kramer multiple comparison post-test, the measurement for 1.0 mM creatine in the presence of 0.2 mM histidine showed a significant difference from the sample without histidine (*p* < 0.001). The presence of other components such as glycine, phenylalanine, tryptophan, and creatinine or the mixture of these components containing histidine had no significant effect on the measurement ability of the creatine-sensing membrane for 1.0 mM creatine (*p* > 0.05) (Figure 5). In addition, no significant interference was observed with the mixture of cations such as Ca^2+^, Na^+^, K^+^, and Mg^2+^ (data not shown).

For biological samples, the ratiometric fluorescence creatine biosensor showed good analytical performance as compared with other creatine biosensors (Table 1). The linear detection range was wide and extended up to 10 mM creatine. The wide linear detection range of the sensing membrane allows quantification of the amounts of creatine in urine samples. The response time of the sensor was relatively fast and its stability was good, attributable to the structure of the supporting materials used for the immobilization of the two enzymes. We speculate the covalent binding between the isocyanate groups in the hydrogel PU and the amine groups of the enzymes, leading to the extension of the shelf-life of the biosensor for several months without any evident loss in the enzyme activity. The ratiometric fluorescent sensing membrane with good sensitivity and long-term stability may be applied to the high-throughput analysis of creatine.

### 3.3. Enzyme Immobilization for the Creatinine-Sensing Membrane

**Figure 6:**
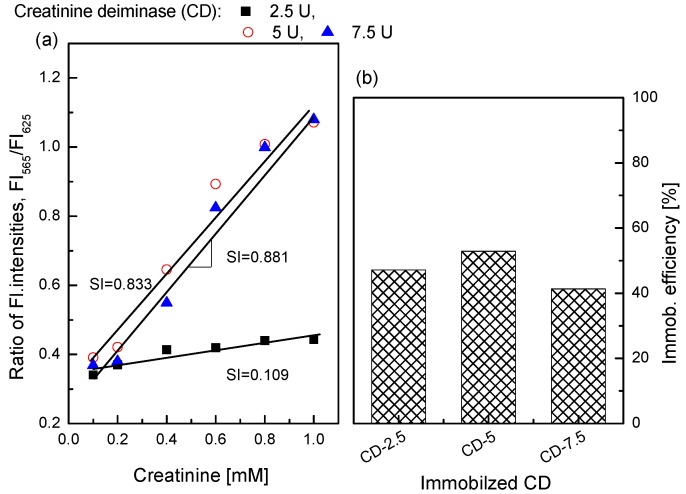
(**a**) The response of the creatinine-sensing membrane immobilized with 2.5, 5, and 7.5 U creatinine deiminase (CD) in the presence of 0.1 to 1.0 mM creatinine. SI represents slope value. (**b**) Immobilization efficiency of various amounts of creatinine deiminase.

The optimal amounts of creatinine deiminase for immobilization into the matrix of EC and PU are tested and shown in Figure 6. The sensing membrane immobilized with 2.5 U creatinine deiminase showed the smallest SI (SI_5U_ = 0.109) in the presence of 0.1–1.0 mM creatinine, whereas SI of the membrane immobilized with 5 or 7.5 U creatinine deiminase (SI_10U_ = 0.833 and SI_15U_ = 0.881) were much higher than those obtained for the membrane immobilized with 2.5 U creatinine deiminase. In addition, data collected from Bradford protein assay indicated that the immobilization efficiency of creatinine deiminase on the second layer of the creatinine-sensing membrane was high at all amounts of creatinine deiminase used. The percentage immobilization was 47.1%, 52.9%, and 41.3% for 2.5, 5, and 7.5 U creatinine deiminase. Based on these observations, 5 U creatinine deiminase was used to catalyze the hydrolysis of creatinine at a concentration range of 0.1 to 1.0 mM to produce ammonia. 

### 3.4. Characterization of the Creatinine-Sensing Membrane

**Figure 7:**
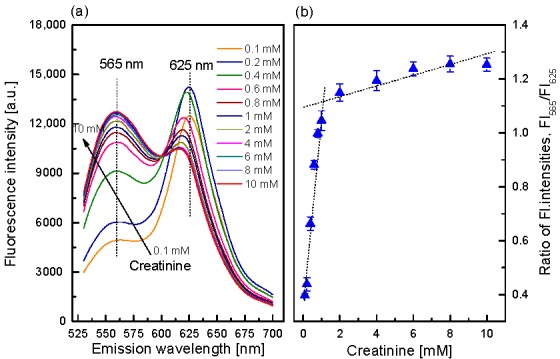
(**a**) Fluorescence emission spectra of the creatinine-sensing membrane in the presence of creatinine at 0.1 to 10 mM concentration monitored at an excitation wavelength of 460 nm. (**b**) Calibration curve for creatinine calculated by the ratio of two fluorescence intensities at emission wavelengths of 565 and 625 nm.

As shown in Figure 7, the linear detection range of creatinine was 0.1–1.0 mM with a high regression coefficient value of *r*^2^ = 0.96, while LOD (S/N = 3) was 0.0325 mM. Given the use of a single enzyme, creatinine deiminase, the response of the creatinine-sensing membrane was similar to that of O17-EC membrane to different ammonia concentrations reported in our previous study [32]. The fluorescence intensity of the creatinine-sensing membrane increased at *λ_em_* = 565 nm and decreased at *λ_em_* = 625 nm in response to an increase in creatinine concentrations from 0.1 to 10 mM. Thus, the sensing membrane with only one enzyme involved in the direct hydrolysis of the analytes to produce ammonia seems to show higher sensitivity and faster response than that with two enzymes. 

The activity of creatinine deiminase immobilized in the matrix of EC and PU was evaluated via Michaelis-Menten kinetics. Kinetic parameters were calculated from the ratio of two emission fluorescence intensities at *λ_em_* = 565 and 625 nm. Lineweaver-Burk plot revealed the maximal reaction rate (V_max_) and Michaelis-Menten constant (K_m_) to be 1.1862 1/min and 22.05 mM, respectively. Studies have shown K_m_ values for free creatinine deiminase to be 1.27 [40] and 0.15 mM [41], while the K_m_ for the creatinine deiminase immobilized on the polyaniline-copper-nanocomposite was calculated to be 0.163 mM [42]. The higher K_m_ value observed in our study may be associated with the very low affinitiy of creatinine deiminase to creatinine in EC and PU matrix as compared with EC-O17 membrane. The low affinity may result from the supporting matrix of EC and PU that was partially bound at the active site of the enzyme, owing to its conformational change. The maximal reaction rate (V_max_ = 0.263 1/min) for the immobilized urease reported in our previous study [32] indicates the strong hydrolysis reaction of creatinine by creatinine deiminase, leading to a short response time of the creatinine-sensing membrane of about t_95_ = 1–3 min at all creatinine concentrations. 

**Figure 8:**
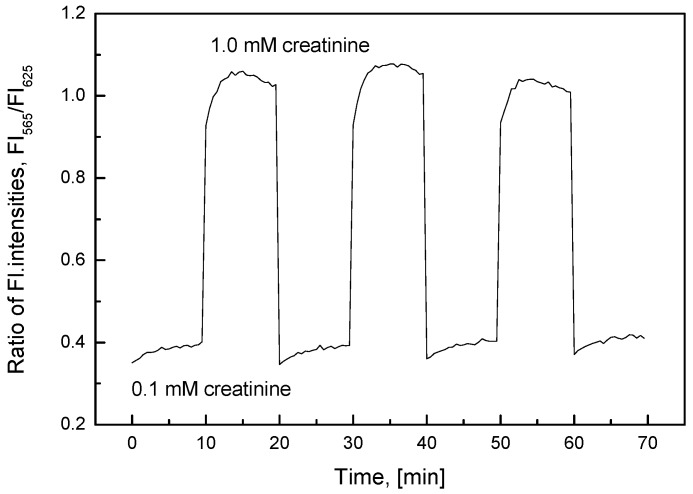
Reversibility of the creatinine-sensing membrane at 0.1 and 1.0 mM creatinine.

Ammonia was probably produced directly through the hydrolysis of creatinine. Therefore, the reproducibility of the creatinine-sensing membrane was rapid, as evident from the repeated exposure of the membrane to 0.1 and 1.0 mM creatinine. Figure 8 shows the reversibility of the creatinine-sensing membrane based on the ratio of the fluorescence intensities at *λ_em_* = 565 and 625 nm. The sensing membrane exhibited a very low relative standard deviation of 1.1% and 1.9% for 0.1 and 1.0 mM creatinine, respectively. 

The creatinine-sensing membrane also preferred an alkaline (pH range of 8.0 to 9.0) to acidic (pH 5.0 to pH 7.0) medium (data not shown). At 0.4 mM creatinine concentration, the ratio of the fluorescence intensities of the creatinine-sensing membrane at *λ_em_* = 565 and 625 nm increased with an increase in the pH to the basic range. The response of the creatinine-sensing membrane at different temperatures was also studied with various creatinine concentrations. The temperature range of 30 to 40 °C failed to exert any significant effect on the sensitivity of the membrane at creatinine concentration of 0.1 to 1.0 mM (*p* = 0.901) (data not shown).

**Figure 9:**
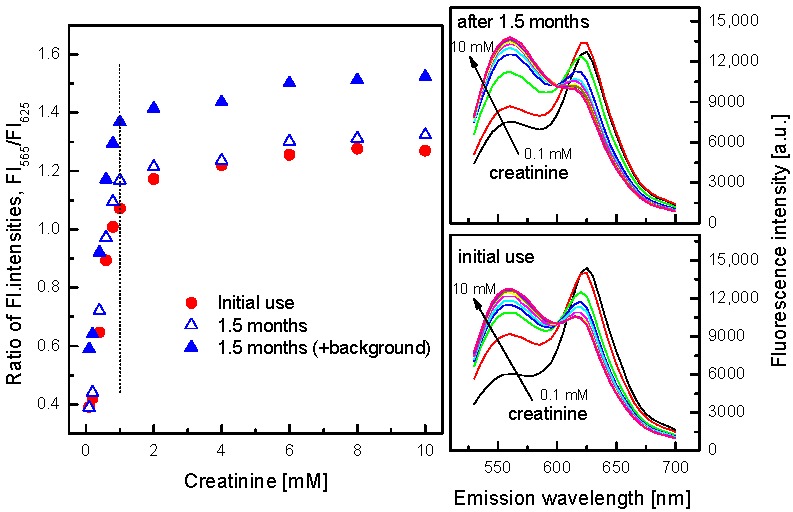
Long-term stability of the creatinine-sensing membrane at 0.1 to 10 mM creatinine concentration upon initial use and after 1.5 months of use (**left**). Two fluorescence emission spectra of the creatinine-sensing membrane with respect to creatinine concentrations at *λ_ex_* = 460 nm.

The long-term stability of the creatinine-sensing membrane was tested after 1.5 months of use and storage. The membrane maintained its high sensitivity to various creatinine concentrations (Figure 9), as evident from the increase in SI of the linear curve at creatinine concentration of 0.1 to 1.0 mM (SI_0.1–1.0 mM_) from 0.832 (initial use) to 0.936 (after 1.5 months). The increase in SI after 1.5 months of use may be related to the good maintenance of the enzyme activity in the polymers EC and PU [39], which created a versatile environment for the enzyme catalysis and response of O17-EC membrane to ammonia produced. In addition, the increase in the background signal of the creatinine-sensing membrane could be recognized after testing the sensing membrane with different pH solutions. This is attributed to the slight leakage of O17 dye upon its exposure to strong pH solutions, thereby increasing the ratio of the fluorescence intensities at *λ_em_* = 565 and 625 nm. The long-term soaking of the polymer in the aqueous solution may have contributed to the increase in the reflection of the incident light and fluorescence emission during creatinine measurements.

**Figure 10:**
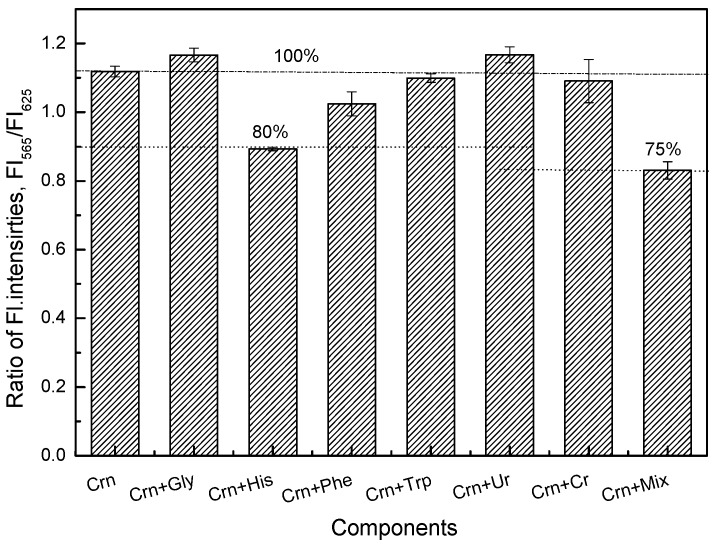
Response of the creatine-sensing membrane to aqueous solutions of 1.0 mM creatinine (Crn) and 0.2 mM of each of the following components: Gly: glycine, His: histidine, Phe: phenylalanine, Trp: tryptophan, Ur: urea, Cr: creatine, and Mix: mixture of Gly, His, Phe, Trp, Ur, and Cr.

The limitation of the spectrometric method is the interference from a few factors in the samples [17,18] that have similar absorption and emission wavelengths or the change in the refractive medium. The influence of some components in urine samples on the creatinine-sensing membrane is shown in Figure 10. According to the results of one-way ANOVA, the presence of 0.2 mM histidine in 1.0 mM creatinine sample resulted in a significant difference in the measurement as compared to samples without histidine (*p* = 0.003). However, the presence of other components such as glycine, phenylalanine, tryptophan, urea, and creatine failed to exert any effect on the measurement of 1.0 mM creatinine (*p* > 0.05); however, the mixture of these components containing histidine also had a significant effect on the creatinine measurement using the creatinine-sensing membrane (*t*-test with *p* = 0.005). No significant interference was observed with a mixture of cations such as Ca^2+^, Na^+^, K^+^, and Mg^2+^ (data not shown). Aside from their influence on the pH of the solution, these cations seemed to have less effect on the response of the creatinine-sensing membrane. Maintaining an alkaline medium during creatinine measurements may guarantee the high sensitivity of the creatinine-sensing membrane.

A few studies have systematically reviewed the techniques of electrochemical enzymic/non-enzymic and immuno-sensors for creatinine detection [17]. Aside from electrochemical transducers, some optical methods based on Jaffe’s reaction or enzyme-catalyzed reactions were also used for the measurement of creatinine. In Table 2, the analytical performance of some optical methods for creatinine detection is compared with that of the ratiometric fluorescence creatinine biosensor developed in this study. The ratiometric fluorescent creatinine biosensor exhibited good analytical performance in terms of high sensitivity, good selectivity, low-cost, and rapidity. In particular, many samples can be analyzed simultaneously using a 96-well microtiter plate with immobilized creatinine-sensing membranes. 

### 3.5. Determination of Creatine and Creatinine in Artificial Urine Solution (AUS)

**Figure 11:**
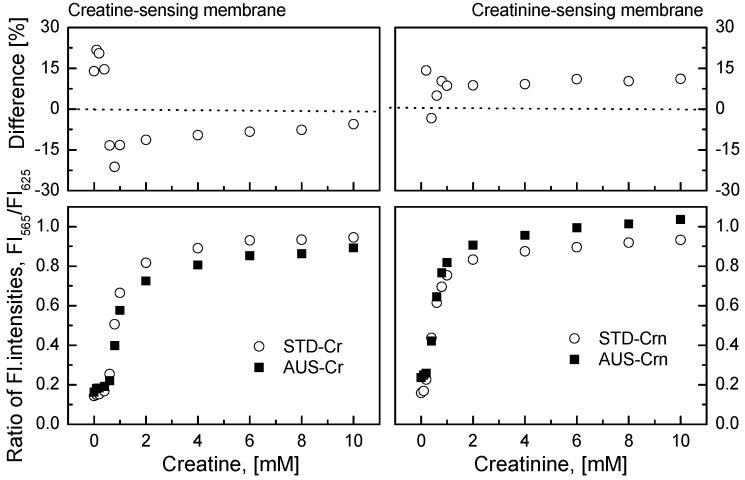
Comparison of creatine (Cr) and creatinine (Crn) concentrations in standard solution (STD) and artificial urine solution (AUS) using creatine- and creatinine-sensing membranes, and their difference plots. The difference represents 100 × (creatine or creatinine in AUS − standard creatine or creatinine)/(standard creatine or creatinine). The dashed lines indicate the recovery percentage of 85–115% for creatine or creatinine in AUS samples.

The creatine- and creatinine-sensing biosensors developed for the detection of creatine and creatinine were used to evaluate their recovery capabilities. The measurement results of creatine and creatinine in the standard solution prepared in 10 mM phosphate buffer saline (pH 7.2) and AUS are shown in Figure 11. The difference plot in Figure 11 also shows the recovery percentage of the concentrations of creatine and creatinine in the AUS. The recovery percentage of two sensing membranes was quite high (85–115%). The large difference in the two measurement results at low concentrations of creatine and creatinine (0–0.2 mM) may be associated with the presence of anions such as SO_4_^2−^ in the AUS. 

## 4. Conclusions

Enzymatic fluorescence assay techniques are highly specific and sensitive, but their use is restricted due to enzyme instability and assay complexity. In this study, ratiometric fluorescent biosensors for creatine and creatinine detection were successfully fabricated. These biosensors showed good sensitivity in the linear concentration range of 0.1–1.0 mM for creatine and 0.1–1.0 mM for creatinine, while their LOD was 0.015 and 0.0325 mM for creatine and creatinine, respectively. The sensing membranes displayed negligible interference from amino acids and salts, with the exception of 0.2 mM histidine. The reproducibility of the sensing membranes for creatine and creatinine was excellent, with a very low relative standard deviation (<2.67%), and their sensitivity to creatine and creatinine was retained for at least 2 months. The high recovery percentage of two sensing membranes in artificial urine samples highlights their potential application for the determination of creatine and creatinine concentrations in clinical chemistry. In addition, the successful development of the ratiometric fluorescent biosensors would be of a great significance in high-throughput screening techniques in analytical biochemistry. 

## Figures and Tables

**Table 1:** Summary of the analytical performance of some creatine biosensors.

Detection Method (Enzymes Used)	Linear Detection Range	Limit of Detection	Response Time	Long-Term Stability	Ref.
Potentiometry (CTN + URS)	0.1–30 mM	10 µM	1.5–4 min	14 days	[5]
Potentiometry (CTN + URS)	0.01–1 mM	10 µM	1–2 min	2 months	[6]
Amperometry (CTN + SOD)	0–2 mM	3.4 µM	3 min	3 months	[7]
Amperometry (CTN + SOD)	0–3.5 mM	n.a.	1 min	8 days	[8]
Amperometry (CTN + SOD)	0.2–3.8 µM and 9–120 µM	0.2 µM	10 s	30 days	[9]
Ratiometric fluorescence (CTN + URS)	0.1–1 Mm and 1–10 mM	0.015 mM	3.5 min	>3 months	This work

CTN: creatinase, SOD: sarcosine oxidase, URS: urease, n.a.: not available.

**Table 2:** Summary of the analytical performance of some optical methods for creatinine detection.

Detection Method (Enzyme or Assay Used)	Linear Detection Range	Limit of Detection	Response Time	Ref.
Light diffraction (CD)	0.01–0.7 mM	6 µM	14 min	[43]
Chemiluminescence (H_2_O_2_-Co^2+^)	0.1–30 µM	0.07 µM	1 min	[44]
Colorimetry (uric acid-Hg^2+^-AuNPs)	1–12 µM	1.6 nM	5 min	[28]
Colorimetry (citrate-AuNPs)	0.1–20 mM	0.72 mM	24 min	[45]
Colorimetry-enzPAD (CNN + CTN + SOD)	0.22–2.2 mM	0.17 mM	11 min	[46]
Fluorescence (QDs-CD conj.)	0.3–5 mM	n.a.	3–4 min	[47]
Ratiometric fluorescence (CD)	0.1–1.0 mM	0.0325 mM	1–3 min	This work

CD: creatinine deiminase, AuNP: gold nanoparticles, CNN: creatininase, CTN: creatinase, SOD: sarcosine oxidase, QD: quantum dots, enz-PAD: enzyme paper-based analytical device.
